# Impact of the COVID‐19 Pandemic on the Incidence, Etiology, Demographics, and Treatment of Craniomaxillofacial Trauma

**DOI:** 10.1002/ohn.981

**Published:** 2024-10-01

**Authors:** F. Jeffrey Lorenz, Andrew J. Rothka, Heather K. Schopper, Jessyka G. Lighthall

**Affiliations:** ^1^ Department of Otolaryngology–Head and Neck Surgery Penn State College of Medicine Hershey Pennsylvania USA; ^2^ College of Medicine The Pennsylvania State University Hershey Pennsylvania USA

**Keywords:** COVID‐19, craniomaxillofacial trauma, facial fractures, facial lacerations, facial soft tissue injuries, facial trauma

## Abstract

**Objective:**

To compare the incidence, etiology, demographics, and treatment of craniomaxillofacial (CMF) trauma before, during, and after COVID‐19.

**Study Design:**

Retrospective cohort.

**Setting:**

Eighty‐three health care organizations across the United States.

**Methods:**

The TriNetX Research Network identified 77,977,880 patients during 2017 to 2022. CMF fractures and soft tissue injuries during March to August of each year, aligning with the 2020 pandemic lockdown, were analyzed.

**Results:**

In 2020, compared to immediately prepandemic in 2019, there were significant reductions of −17.5% in facial fractures and −19.0% in soft tissue injuries (*P* < .001). Conversely, in 2021, both injury types increased by +16.7% and +16.3%, respectively, compared to 2020 (*P* < .001). Changes in injury mechanisms in 2020 included significant decreases in athletic injuries (−57.6%), falls (−16.8%), assaults (−15.5%), motor vehicle collisions (−8.7%), and pedestrian accidents (−6.9%) (*P* < .01), while off‐road vehicle (+48.4%), bicycle (+16.2%), and motorcycle (+8.9%) accidents increased (*P* < .01). The 10‐ to 14‐ and 5‐ to 9‐year‐old age groups experienced the most substantial reductions in facial fractures (−39.7% and −29.9%, respectively) and soft tissue injuries (−29.2% and −28.3%, respectively) in 2020 compared to 2019 (*P* < .001). Operative management of fractures and soft tissue injuries dropped by −20.3% and −12.4%, respectively, in 2020 versus 2019, and then rebounded with +15.8% and +14.6% increases in 2021 compared to 2020 (*P* < .001). In 2022, compared to prepandemic rates of 2019, there were fewer patients with facial fractures (−2.8%), soft tissue injuries (−4.5%), and operative repairs (−6.9% for fractures, −1.2% for soft tissue injuries) (*P* < .03).

**Conclusion:**

CMF trauma decreased in 2020, with subsequent years showing a rebound to levels slightly below those immediately prior to pandemic onset. Changes in etiology, demographics, and treatment highlight the complex dynamics of traumatic injuries during periods of societal disruption.

The COVID‐19 pandemic caused unprecedented societal disruption, affecting individuals and communities. In the United States, measures like stay‐at‐home orders, travel restrictions, curfews, and closures were implemented to curb the virus, disrupting daily routines, work, and social interactions.^1^, ^2^ The health care system also changed significantly,^3^ with elective and nonurgent medical procedures postponed while essential services like emergency and trauma care continued.^4^, ^5^

In the context of trauma, craniomaxillofacial (CMF) injuries are frequently encountered, with assault and motor vehicle collisions (MVCs) being the most common causes.^6^ Studies have shown a reduction in facial trauma during the pandemic, but these are limited to individual or a small number of hospitals and lack large‐scale analysis representative of the entire United States.^7^, ^8^, ^9^, ^10^, ^11^, ^12^, ^13^, ^14^ Furthermore, as society has progressed beyond the peak of the pandemic and embraced the “new normal,” a unique opportunity has arisen to assess postpandemic trends. This research aims to use a comprehensive database from over 80 health care organizations (HCOs) across the United States to investigate the national incidence, causes, demographics, and treatment of facial trauma before, during, and after the pandemic. This will provide valuable insights into how facial trauma patterns have shifted as society moves beyond the pandemic.

## Methods

The data was sourced from the TriNetX Research Network, which includes over 100 million electronic medical records from more than 80 large US HCOs.^15^ The database offers comprehensive information on demographics, diagnoses, procedures, medications, and lab results, supporting large‐scale epidemiological and clinical research. It features advanced analytics for data exploration and cohort analysis, with all data being deidentified and Health Insurance Portability and Accountability Act‐compliant. The study was granted exempt status by the Penn State Institutional Review Board (STUDY00018629).

The TriNetX Research Network was queried using diagnosis (International Classification of Diseases [ICD]‐10) and procedure (Current Procedural Terminology [CPT]) codes to identify individuals interacting with the health care system between 2017 and 2022. CMF fractures and soft tissue injuries of the face, head, or neck were assessed during March to August in these years. Fractures included those of the skull, nasal, orbital, midface, mandible, and other facial bones, while soft tissue injuries covered the scalp, eyelids, nose, ear, cheek, lip, and neck. The March to August timeframe was chosen to reflect the immediate period of stringent lockdown measures in many US states. However, to account for regional and temporal variations in lockdown policies,^16^, ^17^, ^18^ additional analyses were conducted for the full calendar years (January‐December) from 2017 to 2022. CMF fracture and soft tissue injury causes were identified through ICD‐10 codes, including falls, assaults, MVCs, dog bites, pedestrian accidents, bicycle accidents, motorcycle accidents, self‐harm, athletic injuries, and off‐road vehicle incidents. Injury rates were analyzed by patient age, sex, and region, excluding race and ethnicity to avoid stereotyping and limitations in administrative data.^19^, ^20^ Additional analyses covered operative management, categorized by fracture location (eg, nasal bone, mandible, etc) and soft tissue repair type (simple, intermediate, complex, adjacent tissue transfer). Relevant ICD‐10 and CPT codes utilized to conduct the analyses are listed in Supplemental File S1, available online.

### Statistical Analysis

Analyses were conducted on the TriNetX platform using the “Incidence and Prevalence Tool,” which employs Java, R, and Python. Time windows and outcomes were specified with ICD‐10 and CPT codes. The platform enabled real‐time analysis of a patient cohort representative of the general population. For this retrospective cohort study, relative risks, 95% confidence intervals, and associated *P* values were calculated to compare CMF injuries before, during, and after the COVID‐19 pandemic. Data from 2017 to 2022 were analyzed, using 2019 as the prepandemic baseline due to its position as the immediate prepandemic year. Additionally, the transition from the ICD‐9 to ICD‐10 coding classification system in 2015 introduced new coding standards for CMF injuries, which likely took time to fully implement, making 2019 the most reliable prepandemic reference point. Statistical significance was set at *P* < .05.

## Results

### Description of Cohort

**Figure 1:**
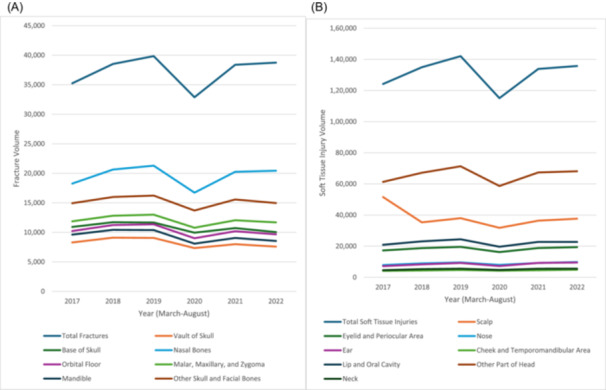
Graphical representation of craniomaxillofacial. (A) Fracture and (B) soft tissue injury volumes during March to August of years before, during, and after the COVID‐19 pandemic (2017‐2022).

A cohort of 77,977,880 patients from 83 HCOs during 2017 to 2022 was identified, representing the general population. Table 1 and Figure 1 show the volumes of CMF fractures and soft tissue injuries from March to August before, during, and after the pandemic. A total of 223,591 patients (0.3%) experienced fractures, and 785,810 (1.0%) had soft tissue injuries of the head, face, or neck.

**Table 1:** CMF Fracture and Soft Tissue Injury Volumes During March to August of Years Before, During, and After the COVID‐19 Pandemic (2017‐2022)

Fracture site	2017	2018 (% Change)	RR (95% CI)	*P* value	2019 (% Change)	RR (95% CI)	*P* value	2020 (% Change)	RR (95% CI)	*P* value	2021 (% Change)	RR (95% CI)	*P* value	2022 (% Change)	RR (95% CI)	*P* value
*CMF fractures*																
Total patients with fractures	35,234	38,517 (+9.3%)	1.09 (1.07‐1.11)	**<.001**	39,844 (+3.4%)	1.03 (1.02‐1.04)	**<.001**	32,880 (−17.5%)	0.83 (0.82‐0.84)	**<.001**	38,379 (+16.7%)	1.17 (1.15‐1.19)	**<.001**	38,737 (+0.9%)	1.01 (1.0‐1.02)	.20
Vault of skull	8281	9099 (+9.9%)	1.10 (1.07‐1.13)	**<.001**	9050 (−0.5%)	0.99 (0.96‐1.02)	.72	7333 (−19.0%)	0.81 (0.79‐0.84)	**<.001**	7988 (+8.9%)	1.09 (1.06‐1.13)	**<.001**	7577 (−5.1%)	0.95 (0.92‐0.98)	**<.001**
Base of skull	10,914	11,710 (+7.3%)	1.07 (1.04‐1.10)	**<.001**	11,655 (−0.5%)	1.0 (0.97‐1.03)	.72	9939 (−14.7%)	0.85 (0.83‐0.87)	**<.001**	10,718 (+7.8%)	1.08 (1.05‐1.11)	**<.001**	10,039 (−6.3%)	0.94 (0.91‐0.97)	**<.001**
Nasal bones	18,251	20,636 (+13.1%)	1.13 (1.11‐1.15)	**<.001**	21,288 (+3.2%)	1.03 (1.01‐1.05)	**.001**	16,722 (−21.4%)	0.79 (0.77‐0.81)	**<.001**	20,252 (+21.1%)	1.21 (1.19‐1.24)	**<.001**	20,430 (+0.9%)	1.01 (0.99‐1.03)	.38
Orbital floor	10,216	11,237 (+10.0%)	1.10 (1.07‐1.13)	**<.001**	11,360 (+1.1%)	1.01 (0.98‐1.04)	.41	8999 (−20.8%)	0.79 (0.77‐0.81)	**<.001**	10,180 (13.1%)	1.13 (1.10‐1.16)	**<.001**	9660 (−5.1%)	0.95 (0.92‐0.98)	**<.001**
Malar, maxillary, and zygoma	11,862	12,811 (+8.0%)	1.08 (1.05‐1.11)	**<.001**	12,991 (+1.4%)	1.01 (0.99‐1.03)	.26	10,780 (−17.0%)	0.83 (0.81‐0.85)	**<.001**	12,042 (+11.7%)	1.12 (1.09‐1.15)	**<.001**	11,680 (−3.0%)	0.97 (0.95‐1.0)	**.02**
Mandible	9614	10,422 (+8.4%)	1.08 (1.05‐1.11)	**<.001**	10,383 (−0.4%)	1.0 (0.97‐1.03)	.79	8083 (−22.2%)	0.78 (0.76‐0.8)	**<.001**	9044 (+11.9%)	1.12 (1.09‐1.15)	**<.001**	8542 (−5.6%)	0.94 (0.91‐0.97)	**<.001**
Other skull and facial bones	14,930	15,985 (+7.0%)	1.07 (1.05‐1.09)	**<.001**	16,226 (+1.5%)	1.02 (1.0‐1.04)	.18	13,692 (−15.6%)	0.84 (0.82‐0.86)	**<.001**	15,561 (+13.7%)	1.14 (1.11‐1.17)	**<.001**	14,955 (−3.9%)	0.96 (0.94‐0.98)	**<.001**
Mean fractures per patient	2.39	2.39 (0%)	‐	‐	2.33 (−2.5%)	‐	‐	2.30 (−1.3%)	‐	‐	2.24 (−2.6%)	‐	**‐**	2.14 (−4.5%)	‐	**‐**
*Facial soft tissue injuries*																
Total patients with soft tissue injuries	124,175	134,912 (+8.6%)	1.09 (1.08‐1.10)	**<.001**	142,057 (+5.3%)	1.05 (1.04‐1.06)	**<.001**	115,086 (−19.0%)	0.81 (0.80‐0.82)	**<.001**	133,851 (+16.3%)	1.16 (1.15‐1.17)	**<.001**	135,729 (+1.4%)	1.01 (1.01‐1.02)	**<.001**
Scalp	51,576	35,320 (−31.5%)	0.68 (0.67‐0.69)	**<.001**	37,976 (+7.5%)	1.08 (1.06‐1.10)	**<.001**	31,793 (−16.3%)	0.84 (0.83‐0.85)	**<.001**	36,373 (+14.4%)	1.14 (1.12‐1.16)	**<.001**	37,649 (+3.5%)	1.04 (1.02‐1.05)	**<.001**
Eyelid and periocular area	17,247	18,737 (+8.6%)	1.09 (1.07‐1.11)	**<.001**	19,546 (+4.3%)	1.04 (1.02‐1.06)	**<.001**	16,210 (−17.1%)	0.83 (0.81‐0.85)	**<.001**	18,792 (+15.9%)	1.16 (1.14‐1.18)	**<.001**	19,402 (+3.2%)	1.03 (1.01‐1.05)	**.002**
Nose	7882	8966 (+13.8%)	1.14 (1.11‐1.18)	**<.001**	9558 (+6.6%)	1.07 (1.04‐1.1)	**<.001**	7974 (−16.6%)	0.83 (0.81‐0.86)	**<.001**	9228 (+15.7%)	1.16 (1.13‐1.2)	**<.001**	9794 (+6.1%)	1.06 (1.03‐1.09)	**<.001**
Ear	7188	8302 (+15.5%)	1.15 (1.11‐1.19)	**<.001**	9231 (+11.2%)	1.11 (1.08‐1.14)	**<.001**	7224 (−21.7%)	0.78 (0.76‐0.80)	**<.001**	9258 (+28.2%)	1.28 (1.24‐1.32)	**<.001**	9492 (+2.5%)	1.03 (1.0‐1.06)	.09
Cheek and temporomandibular area	4149	4393 (+5.9%)	1.06 (1.02‐1.11)	**<.001**	4707 (+7.1%)	1.07 (1.03‐1.11)	**<.001**	4251 (−9.7%)	0.90 (0.86‐0.94)	**<.001**	4544 (+6.9%)	1.07 (1.03‐1.12)	**.002**	4893 (+7.7%)	1.08 (1.03‐1.12)	**<.001**
Lip and oral cavity	20,881	23,095 (+10.6%)	1.11 (1.09‐1.13)	**<.001**	24,433 (+5.8%)	1.06 (1.04‐1.08)	**<.001**	19,632 (−19.6%)	0.80 (0.79‐0.82)	**<.001**	22,750 (+15.9%)	1.16 (1.14‐1.18)	**<.001**	22,724 (−0.1%)	1.0 (0.98‐1.02)	.90
Other part of head	61,290	67,166 (+9.6%)	1.10 (1.09‐1.11)	**<.001**	71,312 (+6.2%)	1.06 (1.05‐1.07)	**<.001**	58,662 (−17.7%)	0.82 (0.81‐0.83)	**<.001**	67,358 (14.8%)	1.15 (1.14‐1.16)	**<.001**	68,133 (+1.2%)	1.01 (1.0‐1.02)	**.04**
Neck	4570	5248 (14.8%)	1.15 (1.11‐1.20)	**<.001**	5544 (+5.6%)	1.06 (1.02‐1.10)	**.004**	4722 (−14.8%)	0.85 (0.82‐0.88)	**<.001**	5530 (+17.1%)	1.17 (1.13‐1.22)	**<.001**	5538 (+0.1%)	1.0 (0.96‐1.04)	.94
Mean soft tissue injuries per patient	1.41	1.27 (−9.9%)	‐	‐	1.28 (+0.8%)	‐	‐	1.31 (+2.3%)	‐	‐	1.30 (−0.8%)	‐	‐	1.31 (+0.8%)	‐	‐

Each RR (95% CI) and *P* value compares the given year to the year prior. Bold values are statistically significant.

Please note individual CMF fracture and soft tissue injury counts exceed total patients with fractures and soft tissue injuries given the fact that some patients had multiple injuries.

Abbreviations: CI, confidence interval; CMF, craniomaxillofacial; RR, relative risk.

### Overall Incidence

From 2017 to 2019, facial fractures increased by +12.7% and soft tissue injuries by +13.9% (both *P* < .001) (Table 1 and Figure 1). In 2020, during the pandemic peak, both declined compared to 2019 (both *P* < .001). In 2021, they increased compared to 2020 (both *P* < .001). By 2022, facial fractures declined by 2.8% and soft tissue injuries by 4.5% compared to 2019 (both *P* < .001).

### Fracture Type/Soft Tissue Injury Location

Nasal bone fractures were the most common, affecting 52.6% of patients with CMF fractures (Table 1 and Figure 1A). All fracture types decreased significantly in 2020 compared to 2019 (all *P* < .001), but increased in 2021 (all *P* < .001). There was a small reduction in fracture volume in 2022 compared to 2019, driven by significant decreases across all fracture locations (all *P* < .001). Patients with CMF fractures averaged 2.29 fractures per person over the entire study period. This average decreased by 8.2% in 2022 compared to 2019.

Soft tissue injuries most commonly affected the scalp (29.4% of patients) (Table 1 and Figure 1B). Like fractures, injuries at all locations decreased significantly from 2019 to 2020 (all *P* < .001), but increased in 2021 (all *P* < .002). The reduction in 2022 compared to 2019 was mainly due to significant decreases in injuries of the lip and oral cavity and other head areas (both *P* < .001). Patients who suffered a soft tissue injury averaged 1.31 soft tissue injuries over the study period.

### Injury Mechanism/Etiology

**Figure 2:**
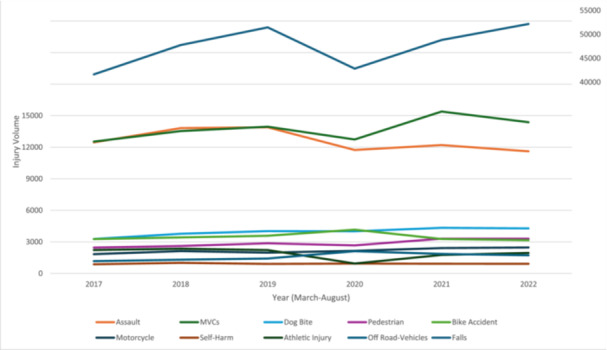
Graphical representation of craniomaxillofacial fracture and soft tissue injury mechanisms during March to August of years before, during, and after the COVID‐19 pandemic (2017‐2022).

Table 2 and Figure 2 detail the causes of CMF fractures and soft tissue injuries. Falls were the predominant cause (mean 47,452 cases per period). During 2020, significant decreases were observed in athletic injuries, falls, assaults, MVCs, and pedestrian accidents compared to 2019 (all *P* < .01), while off‐road vehicle, bicycle, and motorcycle accidents increased (all *P* < .01). Dog bites and self‐harm showed no significant changes (*P* = .90 and *P* = .43, respectively). In 2021, increases in athletic injuries, pedestrian accidents, MVCs, falls, and assaults were noted, with motorcycle accidents continuing to rise (all *P* < .001). Conversely, bicycle and off‐road vehicle accidents decreased (*P* < .001). In 2022, compared to 2019, increases were seen in motorcycle, off‐road vehicle, and pedestrian accidents, dog bites, MVCs, and falls (all *P* < .03), while assaults, bicycle accidents, and athletic injuries decreased (*P* < .001), with no significant change in self‐harm (*P* = .83).

**Table 2:** Craniomaxillofacial Fracture and Soft Tissue Injury Mechanisms During March to August Before, During, and After the COVID‐19 Pandemic (2017‐2022)

Injury mechanism	2017	2018 (% Change)	RR (95% CI)	*P* value	2019 (% Change)	RR (95% CI)	*P* value	2020 (% Change)	RR (95% CI)	*P* value	2021 (% Change)	RR (95% CI)	*P* value	2022 (% Change)	RR (95% CI)	*P* value
Falls	41,630	47,779 (+14.8%)	1.15 (1.13‐1.16)	**<.001**	51,462 (+7.7%)	1.08 (1.06‐1.09)	**<.001**	42,835 (−16.8%)	0.83 (0.82‐0.84)	**<.001**	48,827 (+14.0%)	1.14 (1.13‐1.15)	**<.001**	52,177 (+6.9%)	1.07 (1.06‐1.08)	**<.001**
Assault	12,463	13,821 (+10.9%)	1.11 (1.08‐1.14)	**<.001**	13,892 (+0.5%)	1.01 (0.98‐1.03)	.67	11,735 (−15.5%)	0.84 (0.82‐0.87)	**<.001**	12,206 (+4.0%)	1.04 (1.01‐1.07)	**.002**	11,610 (−4.9%)	0.95 (0.93‐0.98)	**<.001**
MVCs	12,534	13,532 (+8.0%)	1.08 (1.05‐1.11)	**<.001**	13,951 (+3.1%)	1.03 (1.01‐1.06)	**.01**	12,734 (−8.7%)	0.91 (0.89‐0.93)	**<.001**	15,392 (+20.9%)	1.21 (1.18‐1.24)	**<.001**	14,375 (−6.6%)	0.93 (0.91‐0.96)	**<.001**
Dog bite	3280	3773 (+15.0%)	1.15 (1.10‐1.21)	**<.001**	4021 (+6.6%)	1.07 (1.02‐1.11)	**.005**	4010 (−0.3%)	1 (0.95‐1.04)	.90	4337 (+8.2%)	1.08 (1.04‐1.13)	**<.001**	4284 (−1.2%)	0.99 (0.95‐1.03)	.57
Pedestrian	2458	2607 (+6.1%)	1.06 (1.00‐1.12)	**.04**	2865 (+9.9%)	1.10 (1.04‐1.16)	**<.001**	2667 (−6.9%)	0.93 (0.88‐0.98)	**.01**	3308 (+24.0%)	1.24 (1.18‐1.31)	**<.001**	3310 (+0.1%)	1.00 (0.95‐1.05)	.98
Bike accident	3274	3423 (+4.6%)	1.05 (1.00‐1.10)	.07	3580 (+4.6%)	1.05 (1.00‐1.10)	.06	4161 (+16.2%)	1.16 (1.11‐1.22)	**<.001**	3277 (−21.2%)	0.79 (0.75‐0.82)	**<.001**	3163 (−3.5%)	0.97 (0.92‐1.01)	.16
Motorcycle	1835	2125 (+15.8%)	1.16 (1.09‐1.23)	**<.001**	1976 (−7.0%)	0.93 (0.87‐0.99)	**.02**	2152 (+8.9%)	1.09 (1.02‐1.16)	**.01**	2415 (+12.2%)	1.12 (1.06‐1.19)	**<.001**	2466 (+2.1%)	1.02 (0.97‐1.08)	.47
Self‐harm	873	1007 (+15.3%)	1.15 (1.05‐1.26)	**.002**	905 (−10.1%)	0.90 (0.82‐0.98)	**.02**	939 (+3.8%)	1.04 (0.95‐1.14)	.43	922 (−1.8%)	0.98 (0.90‐1.08)	.69	914 (−0.9%)	0.99 (0.90‐1.09)	.85
Athletic injury	2223	2352 (+5.8%)	1.06 (1.00‐1.12)	.06	2224 (−5.4%)	0.95 (0.89‐1.00)	.06	944 (−57.6%)	0.42 (0.39‐0.46)	**<.001**	1759 (+86.3%)	1.86 (1.72‐2.02)	**<.001**	1951 (+10.9%)	1.11 (1.04‐1.18)	**.002**
Off‐road vehicles	1172	1304 (+11.3%)	1.11 (1.03‐1.20)	**.01**	1423 (+9.1%)	1.09 (1.01‐1.18)	**.02**	2112 (+48.4%)	1.48 (1.39‐1.59)	**<.001**	1858 (−12.0%)	0.88 (0.83‐0.94)	**<.001**	1727 (−7.1%)	0.93 (0.87‐0.99)	**.03**

Each RR (95% CI) and *P* value compares the given year to the year prior. Bold values are statistically significant.

Please note mechanism data does not add up to total injuries given the fact that some patients did not have their mechanism coded or had uncommon causes which were not included in the analysis.

Abbreviations: CI, confidence interval; MVC, motor vehicle collision; RR, relative risk.

### Demographic Trends

Table 3 presents CMF fractures by age group. The 20 to 24 and 25 to 29 age brackets were most affected, each comprising 8.2% of all cases from 2017 to 2022. In 2020, all age groups experienced significant reductions compared to 2019 (all *P* < .02), with the largest decrease in 10‐ to 14‐year‐olds, followed by 5 to 9 and 15 to 19. Following these age groups were the more senior 75 to 79, 85 and older, and 80 to 84 year olds. The 40 to 44 age group had the smallest reduction but was still significant. In 2021, all age groups except 50 to 54 (*P* = .10) and 55 to 59 (*P *= .38) saw significant increases compared to 2020 (all *P* < .002), with the 10 to 14 and 85+ age groups showing the most notable rises. In 2022, reductions compared to 2019 were significant in the 5 to 9, 10 to 14, 20 to 24, 25 to 29, 45 to 49, 50 to 54, and 55 to 59 age groups (all *P* < .04), while increases were observed in the 65 to 69, 70 to 74, and 75 to 79 groups (all *P* < .02), with no significant changes in other groups (all *P* > .05).

**Table 3:** Craniomaxillofacial Fracture Volumes During March to August of Years Before, During, and After the COVID‐19 Pandemic (2017‐2022), Stratified by Age

Age	2017	2018 (% Change)	RR (95% CI)	*P* value	2019 (% Change)	RR (95% CI)	*P* value	2020 (% Change)	RR (95% CI)	*P* value	2021 (% Change)	RR (95% CI)	*P* value	2022 (% Change)	RR (95% CI)	*P* value
0‐4	1840	1956 (+6.3%)	1.06 (0.99‐1.13)	.06	2056 (+5.1%)	1.05 (0.99‐1.12)	.11	1758 (−14.5%)	0.86 (0.81‐0.92)	**<.001**	1953 (+11.1%)	1.11 (1.04‐1.18)	**.001**	2037 (+4.3%)	1.04 (0.98‐1.11)	.18
5‐9	1214	1312 (+8.1%)	1.08 (1.0‐1.17)	**.05**	1305 (−0.5%)	0.99 (0.92‐1.07)	.89	915 (−29.9%)	0.70 (0.64‐0.76)	**<.001**	1119 (+22.3%)	1.22 (1.12‐1.33)	**<.001**	1132 (+1.2%)	1.01 (0.93‐1.10)	.78
10‐14	2039	2108 (+3.4%)	1.03 (0.97‐1.09)	.28	2143 (+1.7%)	1.02 (0.96‐1.08)	.59	1293 (−39.7%)	0.60 (0.56‐0.64)	**<.001**	1988 (+53.8%)	1.54 (1.44‐1.65)	**<.001**	2013 (+1.3%)	1.01 (0.95‐1.08)	.69
15‐19	2964	3016 (+1.8%)	1.02 (0.97‐1.07)	.50	3134 (+3.9%)	1.04 (0.99‐1.09)	.13	2403 (−23.3%)	0.77 (0.73‐0.81)	**<.001**	2881 (+19.9%)	1.20 (1.14‐1.27)	**<.001**	3020 (+4.8%)	1.05 (1.0‐1.10)	.07
20‐24	3350	3316 (−1.0%)	0.99 (0.94‐1.04)	.68	3138 (−5.4%)	0.95 (0.90‐1.0)	**.03**	2702 (−13.9%)	0.86 (0.82‐0.91)	**<.001**	3031 (+12.2%)	1.12 (1.06‐1.18)	**<.001**	2861 (−5.6%)	0.94 (0.90‐0.99)	**.03**
25‐29	3045	3243 (+6.5%)	1.07 (1.02‐1.12)	**.01**	3317 (+2.3%)	1.02 (0.97‐1.07)	.36	2751 (−17.1%)	0.83 (0.79‐0.87)	**<.001**	3063 (+11.3%)	1.11 (1.05‐1.17)	**<.001**	2877 (−6.1%)	0.94 (0.89‐0.99)	**.02**
30‐34	2599	2815 (+8.3%)	1.08 (1.02‐1.14)	**.003**	2919 (+3.7%)	1.04 (0.99‐1.10)	.17	2599 (−11.0%)	0.89 (0.84‐0.94)	**<.001**	3003 (+15.5%)	1.16 (1.10‐1.22)	**<.001**	2970 (−1.1%)	0.99 (0.94‐1.04)	.67
35‐39	2252	2595 (+15.2%)	1.15 (1.09‐1.22)	**<.001**	2677 (+3.2%)	1.03 (0.98‐1.09)	.26	2184 (−18.4%)	0.82 (0.77‐0.87)	**<.001**	2607 (+19.4%)	1.19 (1.12‐1.26)	**<.001**	2587 (−0.8%)	0.99 (0.94‐1.05)	.78
40‐44	1846	2007 (+8.7%)	1.09 (1.02‐1.16)	**.01**	2164 (+7.8%)	1.08 (1.02‐1.15)	**.02**	2011 (−7.1%)	0.93 (0.88‐0.99)	**.02**	2237 (+11.2%)	1.11 (1.05‐1.18)	**.001**	2282 (+2.0%)	1.02 (0.96‐1.08)	.50
45‐49	2000	2059 (+3.0%)	1.03 (0.97‐1.10)	.35	2132 (+3.5%)	1.04 (0.98‐1.10)	.26	1765 (−17.2%)	0.83 (0.78‐0.88)	**<.001**	1960 (+11.0%)	1.11 (1.04‐1.18)	**.001**	1846 (−5.8%)	0.94 (0.88‐1.0)	.06
50‐54	2122	2275 (+7.2%)	1.07 (1.01‐1.14)	**.02**	2185 (−4.0%)	0.96 (0.91‐1.02)	.18	1850 (−15.3%)	0.85 (0.80‐0.90)	**<.001**	1951 (+5.5%)	1.05 (0.99‐1.12)	.10	1940 (−0.6%)	0.99 (0.93‐1.06)	.86
55‐59	2077	2321 (+11.7%)	1.12 (1.06‐1.19)	**<.001**	2429 (+4.7%)	1.05 (0.99‐1.11)	.12	2116 (−12.9%)	0.87 (0.82‐0.92)	**<.001**	2174 (+2.7%)	1.03 (0.97‐1.09)	.38	2098 (−3.5%)	0.97 (0.91‐1.02)	.24
60‐64	1820	1965 (+8.0%)	1.08 (1.01‐1.15)	**.02**	2101 (+6.9%)	1.07 (1.01‐1.14)	**.03**	1911 (−9.0%)	0.91 (0.86‐0.97)	**.003**	2184 (+14.3%)	1.14 (1.07‐1.21)	**<.001**	2166 (−0.8%)	0.99 (0.93‐1.05)	.78
65‐69	1479	1836 (+24.1%)	1.24 (1.16‐1.33)	**<.001**	1889 (+2.9%)	1.03 (0.97‐1.10)	.39	1576 (−16.6%)	0.83 (0.78‐0.89)	**<.001**	1928 (+22.3%)	1.22 (1.14‐1.30)	**<.001**	2039 (+5.8%)	1.06 (0.99‐1.13)	.08
70‐74	1216	1587 (+30.5%)	1.31 (1.22‐1.41)	**<.001**	1669 (+5.2%)	1.05 (0.98‐1.12)	.15	1452 (−13.0%)	0.87 (0.81‐0.93)	**<.001**	1812 (+24.8%)	1.25 (1.17‐1.34)	**<.001**	2000 (+10.4%)	1.10 (1.04‐1.18)	**.002**
75‐79	1087	1362 (+25.3%)	1.25 (1.15‐1.35)	**<.001**	1594 (+17.0%)	1.17 (1.09‐1.26)	**<.001**	1235 (−22.5%)	0.77 (0.71‐0.83)	**<.001**	1525 (+23.5%)	1.23 (1.14‐1.33)	**<.001**	1736 (+13.8%)	1.14 (1.06‐1.22)	**<.001**
80‐84	1118	1298 (+16.1%)	1.16 (1.07‐1.26)	**<.001**	1356 (+4.5%)	1.04 (0.96‐1.12)	.26	1077 (−20.6%)	0.79 (0.73‐0.86)	**<.001**	1267 (+17.6%)	1.18 (1.09‐1.28)	**<.001**	1412 (+11.4%)	1.11 (1.03‐1.20)	**.005**
85 and older	1166	1446 (+24.0%)	1.24 (1.15‐1.34)	**<.001**	1636 (+13.1%)	1.13 (1.05‐1.21)	**<.001**	1282 (−21.6%)	0.78 (0.73‐0.84)	**<.001**	1696 (+32.3%)	1.32 (1.23‐1.42)	**<.001**	1721 (+1.5%)	1.01 (0.95‐1.09)	.67

Each RR (95% CI) and *P* value compares the given year to the year prior. Bold values are statistically significant.

Abbreviations: CI, confidence interval; RR, relative risk.

Table 4 outlines soft tissue injuries by age group. Children aged 0 to 4 had the highest incidence, accounting for 18.7% of cases. In 2020, all age groups saw significant decreases (all *P* < .001), with the most substantial declines in 10 to 14, 5 to 9, and 15 to 19 year‐olds. The 80 to 84, 75 to 79, and 85+ age groups also had notable decreases. In 2021, all age groups showed significant increases compared to 2020 (all *P* < .002), especially 70 to 74 and 75 to 79 year‐olds. In 2022, reductions compared to 2019 were significant in the 0 to 4, 5 to 9, 10 to 14, 15 to 19, 20 to 24, 25 to 29, 35 to 39, 45 to 49, 50 to 54, and 55 to 59 age groups (all *P* < .05). Conversely, increases were observed in the 40 to 44, 60 to 64, 65 to 69, 70 to 74, 75 to 79, and 80 to 84 age groups (all *P* < .02), with no significant changes in other groups (all *P* > .05).

**Table 4:** Craniomaxillofacial Soft Tissue Injury Volumes During March to August of Years Before, During, and After the COVID‐19 Pandemic (2017‐2022), Stratified by Age

Age	2017	2018 (% Change)	RR (95% CI)	*P* value	2019 (% Change)	RR (95% CI)	*P* value	2020 (% Change)	RR (95% CI)	*P* value	2021 (% Change)	RR (95% CI)	*P* value	2022 (% Change)	RR (95% CI)	*P* value
0‐4	24,788	26,771 (+8.0%)	1.08 (1.06‐1.10)	**<.001**	27,001 (+0.9%)	1.01 (0.99‐1.03)	.32	21,213 (−21.4%)	0.79 (0.78‐0.80)	**<.001**	24,317 (+14.6%)	1.15 (1.13‐1.17)	**<.001**	22,955 (−5.6%)	0.94 (0.93‐0.96)	**<.001**
5‐9	13,434	14,116 (+5.1%)	1.05 (1.03‐1.08)	**<.001**	14,648 (+3.8%)	1.04 (1.02‐1.06)	**.002**	10,506 (−28.3%)	0.72 (0.70‐0.74)	**<.001**	12,469 (+18.7%)	1.19 (1.16‐1.22)	**<.001**	12,696 (+1.8%)	1.02 (0.99‐1.04)	.15
10‐14	6091	6253 (+2.7%)	1.03 (0.99‐1.07)	.14	6854 (+9.6%)	1.10 (1.06‐1.14)	**<.001**	4854 (−29.2%)	0.71 (0.68‐0.74)	**<.001**	5601 (+15.4%)	1.15 (1.11‐1.20)	**<.001**	6414 (+14.5%)	1.15 (1.10‐1.19)	**<.001**
15‐19	7136	7481 (+4.8%)	1.05 (1.02‐1.08)	**.004**	8017 (+7.2%)	1.07 (1.04‐1.1)	**<.001**	6215 (−22.5%)	0.78 (0.75‐0.81)	**<.001**	7468 (+20.2%)	1.20 (1.16‐1.24)	**<.001**	7769 (+4.0%)	1.04 (1.01‐1.07)	**.01**
20‐24	8070	8175 (+1.3%)	1.01 (0.98‐1.04)	.41	8455 (+3.4%)	1.03 (1.0‐1.06)	**.03**	7155 (−15.4%)	0.85 (0.82‐0.88)	**<.001**	8159 (+14.0%)	1.14 (1.10‐1.18)	**<.001**	7936 (−2.7%)	0.97 (0.94‐1.01)	.08
25‐29	7414	7831 (+5.6%)	1.06 (1.03‐1.09)	**<.001**	8118 (+3.7%)	1.04 (1.01‐1.07)	**.02**	6890 (−15.1%)	0.85 (0.82‐0.88)	**<.001**	7743 (+12.4%)	1.12 (1.08‐1.16)	**<.001**	7436 (−4.0%)	0.96 (0.93‐0.99)	**.01**
30‐34	6052	6690 (+10.5%)	1.11 (1.07‐1.15)	**<.001**	6967 (+4.1%)	1.04 (1.01‐1.08)	**.02**	6228 (−10.6%)	0.89 (0.86‐0.92)	**<.001**	7240 (+16.2%)	1.16 (1.12‐1.20)	**<.001**	7054 (−2.6%)	0.97 (0.94‐1.01)	.12
35‐39	5243	5735 (+9.4%)	1.09 (1.05‐1.13)	**<.001**	6206 (+8.2%)	1.08 (1.04‐1.12)	**<.001**	5353 (−13.7%)	0.86 (0.83‐0.89)	**<.001**	6097 (+13.9%)	1.14 (1.10‐1.18)	**<.001**	5947 (−2.5%)	0.98 (0.94‐1.01)	.17
40‐44	4382	4894 (+11.7%)	1.12 (1.08‐1.17)	**<.001**	5111 (+4.4%)	1.04 (1.0‐1.08)	**.03**	4581 (−10.4%)	0.90 (0.86‐0.94)	**<.001**	5352 (+16.8%)	1.17 (1.12‐1.22)	**<.001**	5462 (+2.1%)	1.02 (0.98‐1.06)	.29
45‐49	4631	5015 (+8.3%)	1.08 (1.04‐1.12)	**<.001**	5196 (+3.6%)	1.04 (1.0‐1.08)	.07	4349 (−16.3%)	0.84 (0.81‐0.87)	**<.001**	4644 (+6.8%)	1.07 (1.03‐1.12)	**.002**	4561 (−1.8%)	0.98 (0.94‐1.02)	.39
50‐54	5095	5366 (+5.3%)	1.05 (1.01‐1.09)	**.008**	5308 (−1.1%)	0.99 (0.95‐1.03)	.57	4522 (−14.8%)	0.85 (0.82‐0.88)	**<.001**	4997 (+10.5%)	1.11 (1.07‐1.16)	**<.001**	4948 (−1.0%)	0.99 (0.95‐1.03)	.62
55‐59	5400	5794 (+7.3%)	1.07 (1.03‐1.11)	**<.001**	6220 (+7.4%)	1.07 (1.03‐1.11)	**<.001**	5152 (−17.2%)	0.83 (0.80‐0.86)	**<.001**	5594 (+8.6%)	1.09 (1.05‐1.13)	**<.001**	5547 (−0.8%)	0.99 (0.96‐1.03)	.66
60‐64	4855	5487 (+13.0%)	1.13 (1.09‐1.17)	**<.001**	5772 (+5.2%)	1.05 (1.01‐1.09)	**.007**	5048 (−12.5%)	0.87 (0.84‐0.9)	**<.001**	5833 (+15.6%)	1.16 (1.12‐1.2)	**<.001**	6031 (+3.4%)	1.03 (1.0‐1.07)	.07
65‐69	4571	5282 (+15.6%)	1.16 (1.11‐1.21)	**<.001**	5674 (+7.4%)	1.07 (1.03‐1.11)	**<.001**	4871 (−14.2%)	0.86 (0.83‐0.89)	**<.001**	5783 (+18.7%)	1.19 (1.15‐1.24)	**<.001**	6253 (+8.1%)	1.08 (1.04‐1.12)	**<.001**
70‐74	4122	4864 (+18.0%)	1.18 (1.13‐1.23)	**<.001**	5385 (+10.7%)	1.11 (1.07‐1.15)	**<.001**	4501 (−16.4%)	0.84 (0.81‐0.87)	**<.001**	5906 (+31.2%)	1.31 (1.26‐1.36)	**<.001**	6382 (+8.1%)	1.08 (1.04‐1.12)	**<.001**
75‐79	3745	4428 (+18.2%)	1.18 (1.13‐1.23)	**<.001**	5262 (+18.8%)	1.19 (1.14‐1.24)	**<.001**	4208 (−20.0%)	0.80 (0.77‐0.83)	**<.001**	5366 (+27.5%)	1.28 (1.23‐1.33)	**<.001**	6018 (+12.2%)	1.12 (1.08‐1.16)	**<.001**
80‐84	4012	4522 (+12.7%)	1.13 (1.08‐1.18)	**<.001**	4897 (+8.3%)	1.08 (1.04‐1.12)	**<.001**	3812 (−22.2%)	0.78 (0.75‐0.81)	**<.001**	4574 (+20.0%)	1.20 (1.15‐1.25)	**<.001**	5145 (+12.5%)	1.12 (1.08‐1.17)	**<.001**
85 and older	5134	6208 (+20.9%)	1.21 (1.17‐1.26)	**<.001**	6966 (+12.2%)	1.12 (1.08‐1.16)	**<.001**	5628 (−19.2%)	0.81 (0.78‐0.84)	**<.001**	6708 (+19.2%)	1.19 (1.15‐1.23)	**<.001**	7175 (+7.0%)	1.07 (1.03‐1.11)	**<.001**

Each RR (95% CI) and *P* value compares the given year to the year prior. Bold values are statistically significant.

Abbreviations: CI, confidence interval; RR, relative risk.

Table 5 shows facial fractures and soft tissue injuries by gender. Males consistently had 69.4% more fractures and 56.1% more soft tissue injuries than females. Both genders saw significant reductions in fractures and soft tissue injuries in 2020 compared to 2019 (all *P* < .001). In 2021, both experienced significant increases (all *P* < .001), with females showing a slightly greater rise. By 2022, males had a significant decrease in fractures compared to 2019 (*P* < .001), while females showed no significant change (*P* = .26). Both genders had significant reductions in soft tissue injuries during the same period (*P* < .001).

**Table 5:** Craniomaxillofacial Fracture and Soft Tissue Injury Volumes During March to August of Years Before, During, and After the COVID‐19 Pandemic (2017‐2022), Stratified by Sex

Sex	2017	2018 (% Change)	RR (95% CI)	*P* value	2019 (% Change)	RR (95% CI)	*P* value	2020 (% Change)	RR (95% CI)	*P* value	2021 (% Change)	RR (95% CI)	*P* value	2022 (% Change)	RR (95% CI)	*P* value
*Craniomaxillofacial fractures*																
Female	12,014	13,506 (+12.4%)	1.12 (1.09‐1.15)	**<.001**	13,951 (+3.3%)	1.03 (1.01‐1.05)	**.007**	11,444 (−18.0%)	0.82 (0.80‐0.84)	**<.001**	13,807 (+20.6%)	1.21 (1.18‐1.24)	**<.001**	14,139 (+2.4%)	1.02 (1.0‐1.05)	**.05**
Male	21,681	23,040 (+6.3%)	1.06 (1.04‐1.08)	**<.001**	23,822 (+3.4%)	1.03 (1.02‐1.05)	**<.001**	19,887 (−16.5%)	0.83 (0.82‐0.85)	**<.001**	22,550 (+13.4%)	1.13 (1.11‐1.16)	**<.001**	22,669 (+0.5%)	1.01 (0.99‐1.02)	.58
Unknown	1539	1971 (+28.1%)	1.28 (1.20‐1.37)	**<.001**	2071 (+5.1%)	1.05 (0.99‐1.12)	.12	1549 (−25.2%)	0.75 (0.70‐0.80)	**<.001**	2022 (+30.5%)	1.31 (1.23‐1.40)	**<.001**	1929 (−4.6%)	0.95 (0.90‐1.02)	.14
*Facial soft tissue injuries*																
Female	46,200	51,010 (+10.4%)	1.10 (1.09‐1.11)	**<.001**	54,258 (+6.4%)	1.06 (1.05‐1.07)	**<.001**	43,844 (−19.2%)	0.81 (0.80‐0.82)	**<.001**	51,866 (+18.3%)	1.18 (1.17‐1.20)	**<.001**	52,644 (+1.5%)	1.02 (1.0‐1.03)	**.02**
Male	76,196	81,203 (+6.6%)	1.07 (1.06‐1.08)	**<.001**	84,420 (+4.0%)	1.04 (1.03‐1.05)	**<.001**	68,514 (−18.5%)	0.81 (0.80‐0.82)	**<.001**	78,369 (+14.4%)	1.14 (1.13‐1.15)	**<.001**	79,305 (+1.2%)	1.01 (1.0‐1.02)	**.02**
Unknown	1779	2699 (+51.7%)	1.52 (1.43‐1.61)	**<.001**	3379 (+25.2%)	1.25 (1.19‐1.31)	**<.001**	2728 (−19.3%)	0.81 (0.77‐0.85)	**<.001**	3616 (+32.6%)	1.33 (1.27‐1.40)	**<.001**	3780 (+4.5%)	1.05 (1.0‐1.09)	.06

Each RR (95% CI) and *P* value compares the given year to the year prior. Bold values are statistically significant.

Abbreviations: CI, confidence interval; RR, relative risk.

### Treatment

**Figure 3:**
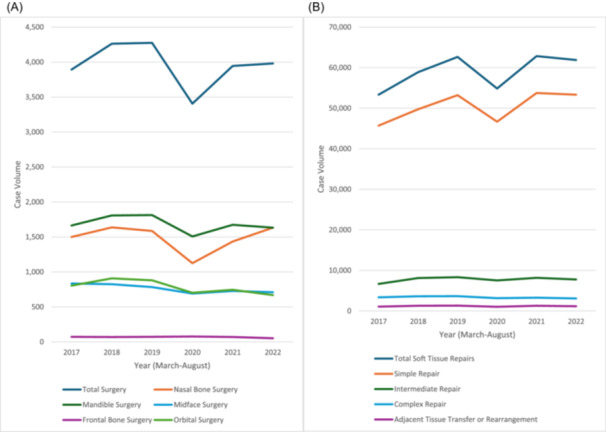
Graphical representation of craniomaxillofacial. (A) Fracture and (B) soft tissue injury operative repair volumes during March to August of years before, during, and after the COVID‐19 pandemic (2017‐2022).

Table 6 and Figure 3 outline trends in operative management. Fracture surgeries were performed in 23,762 cases (10.6% of all fracture patients), with 42.5% involving mandibular surgery. For soft tissue injuries, 354,456 surgeries were conducted (45.1% of patients), with 85.3% being simple repairs. Fracture surgeries declined in 2020 and rebounded in 2021 (both *P* < .001). Except for frontal bone surgery (*P* = .68), all surgery types significantly decreased in 2020 (all *P* < .02), with nasal bone surgeries showing the largest fluctuation. Soft tissue injury surgeries also dropped in 2020 and recovered in 2021 (both *P* < .001). By 2022, fracture surgeries had decreased compared to 2019 (*P* < .001), with a slight reduction in soft tissue repairs (*P* = .03). Patients with fractures who underwent surgery had an average of 1.21 operative fracture repairs, while those with soft tissue injuries averaged 1.06 repairs.

**Table 6:** Craniomaxillofacial Fracture and Soft Tissue Injury Operative Repair Volumes During March to August of Years Before, During, and After the COVID‐19 Pandemic (2017‐2022)

Surgery type	2017	2018 (% Change)	RR (95% CI)	*P* value	2019 (% Change)	RR (95% CI)	*P* value	2020 (% Change)	RR (95% CI)	*P* value	2021 (% Change)	RR (95% CI)	*P* value	2022 (% Change)	RR (95% CI)	*P* value
*Craniomaxillofacial fracture operative repairs*																
Total patients with surgery	3893	4263 (+9.5%)	1.10 (1.05‐1.15)	**<.001**	4275 (+0.3%)	1.0 (0.96‐1.04)	.90	3406 (−20.3%)	0.80 (0.76‐0.84)	**<.001**	3945 (+15.8%)	1.16 (1.11‐1.21)	**<.001**	3980 (+0.9%)	1.01 (0.97‐1.05)	.69
Nasal bone surgery	1500	1637 (+9.1%)	1.09 (1.02‐1.17)	**.01**	1586 (−3.1%)	0.97 (0.91‐1.04)	.37	1125 (−29.1%)	0.71 (0.66‐0.77)	**<.001**	1435 (+27.6%)	1.28 (1.18‐1.38)	**<.001**	1633 (+13.8%)	1.14 (1.06‐1.22)	**<.001**
Mandible surgery	1664	1808 (+8.7%)	1.09 (1.02‐1.17)	**.02**	1813 (+0.3%)	1.0 (0.94‐1.07)	.93	1507 (−16.9%)	0.83 (0.78‐0.89)	**<.001**	1674 (+11.1%)	1.11 (1.04‐1.19)	**.003**	1633 (−2.4%)	0.98 (0.91‐1.04)	.48
Midface surgery	835	824 (−1.3%)	0.99 (0.90‐1.09)	.79	783 (−5.0%)	0.95 (0.86‐1.05)	.31	691 (−11.7%)	0.88 (0.79‐0.97)	**.02**	728 (+5.4%)	1.05 (0.95‐1.17)	.33	710 (−2.5%)	0.98 (0.88‐1.08)	.64
Frontal bone surgery	72	69 (−4.2%)	0.96 (0.69‐1.34)	.80	72 (+4.3%)	1.04 (0.75‐1.45)	.80	77 (+6.9%)	1.07 (0.78‐1.48)	.68	70 (−9.1%)	0.91 (0.66‐1.26)	.56	52 (−25.7%)	0.74 (0.52‐1.06)	.10
Orbital surgery	804	908 (+12.9%)	1.13 (1.03‐1.24)	**.01**	880 (−3.1%)	0.97 (0.88‐1.06)	.51	702 (−20.2%)	0.80 (0.72‐0.88)	**<.001**	745 (+6.1%)	1.06 (0.96‐1.18)	.26	669 (−10.2%)	0.90 (0.81‐1.0)	**.04**
Mean procedures per patient	1.25	1.23 (−1.6%)	‐	‐	1.20 (−2.4%)	‐	‐	1.20 (0%)	‐	‐	1.18 (−1.7%)	‐	‐	1.18 (0%)	‐	**‐**
*Facial soft tissue repairs*																
Total patients with soft tissue repair	53,336	58,887 (+10.4%)	1.10 (1.09‐1.11)	**<.001**	62,648 (+6.4%)	1.06 (1.05‐1.07)	**<.001**	54,852 (−12.4%)	0.88 (0.87‐0.89)	**<.001**	62,850 (+14.6%)	1.15 (1.14‐1.16)	**<.001**	61,883 (−1.5%)	0.98 (0.97‐1.0)	**.006**
Simple repair	45,708	49,739 (+8.8%)	1.09 (1.08‐1.10)	**<.001**	53,211 (+7.0%)	1.07 (1.06‐1.08)	**<.001**	46,670 (−12.3%)	0.88 (0.87‐0.89)	**<.001**	53,746 (+15.2%)	1.15 (1.14‐1.16)	**<.001**	53,333 (−0.8%)	0.99 (0.98‐1.0)	.21
Intermediate repair	6666	8112 (+21.7%)	1.22 (1.18‐1.26)	**<.001**	8329 (+2.7%)	1.03 (1.0‐1.06)	.09	7520 (−9.7%)	0.90 (0.87‐0.93)	**<.001**	8161 (+8.5%)	1.09 (1.06‐1.12)	**<.001**	7763 (−4.9%)	0.95 (0.92‐0.98)	**.002**
Complex repair	3358	3608 (+7.4%)	1.07 (1.02‐1.12)	**.003**	3645 (+1.0%)	1.01 (0.96‐1.06)	.66	3146 (−13.7%)	0.86 (0.82‐0.90)	**<.001**	3271 (+4.0%)	1.04 (0.99‐1.09)	.12	3082 (−5.8%)	0.94 (0.90‐0.99)	**.02**
Adjacent tissue transfer or rearrangement	1064	1279 (+20.2%)	1.20 (1.11‐1.30)	**<.001**	1312 (+2.6%)	1.03 (0.95‐1.11)	.52	1023 (−22.0%)	0.78 (0.72‐0.85)	**<.001**	1283 (+25.4%)	1.25 (1.15‐1.36)	**<.001**	1161 (−9.5%)	0.90 (0.84‐0.98)	**.01**
Mean procedures per patient	1.06	1.07 (+0.9%)	‐	‐	1.06 (−0.9%)	‐	‐	1.06 (0%)	‐	‐	1.06 (0%)	‐	‐	1.06 (0%)	‐	‐

Each RR (95% CI) and *P* value compares the given year to the year prior. Bold values are statistically significant.

Please note individual surgeries and repairs exceed total patients with surgery or soft tissue repairs given the fact that some patients had multiple injuries repaired.

Abbreviations: CI, confidence interval; RR, relative risk.

### Regional Data

Table 7 and Supplemental File S9, available online display regional facial fracture and soft tissue injury volumes. In 2020, all regions saw significant decreases compared to 2019 (*P* < .001), with the Midwest and West showing the smallest magnitude of decline.

**Table 7:** Craniomaxillofacial Fracture and Soft Tissue Injury Volumes During March to August of Years Before, During, and After the COVID‐19 Pandemic (2017‐2022), Stratified by Region

Region	2017	2018 (% Change)	RR (95% CI)	*P* value	2019 (% Change)	RR (95% CI)	*P* value	2020 (% Change)	RR (95% CI)	*P* value	2021 (% Change)	RR (95% CI)	*P* value	2022 (% Change)	RR (95% CI)	*P* value
*Craniomaxillofacial fractures*																
Northeast	10,886	12,865 (+18.2%)	1.18 (1.15‐1.21)	**<.001**	13,667 (+6.2%)	1.06 (1.04‐1.09)	**<.001**	10,812 (−20.9%)	0.79 (0.77‐0.81)	**<.001**	12,979 (+20.0%)	1.20 (1.17‐1.23)	**<.001**	13,769 (+6.1%)	1.06 (1.04‐1.09)	**<.001**
Midwest	6086	6088 (0%)	1.00 (0.97‐1.04)	.99	6219 (+2.2%)	1.02 (0.99‐1.06)	.24	5759 (−7.4%)	0.93 (0.89‐0.96)	**<.001**	6419 (+11.5%)	1.11 (1.08‐1.16)	**<.001**	5957 (−7.2%)	0.93 (0.90‐0.96)	**<.001**
South	10,737	11,471 (+6.8%)	1.07 (1.04‐1.10)	**<.001**	11,877 (+3.5%)	1.04 (1.01‐1.06)	**.008**	9599 (−19.2%)	0.81 (0.79‐0.83)	**<.001**	11,277 (+17.5%)	1.17 (1.14‐1.21)	**<.001**	11,650 (+3.3%)	1.03 (1.01‐1.06)	**.01**
West	4412	5043 (+14.3%)	1.14 (1.10‐1.19)	**<.001**	5268 (+4.5%)	1.04 (1.01‐1.09)	**.03**	4549 (−13.6%)	0.86 (0.83‐0.90)	**<.001**	5287 (+16.2%)	1.16 (1.12‐1.21)	**<.001**	5286 (0%)	1.0 (0.96‐1.04)	.99
Other/unknown	3113	3050 (−3.0%)	0.98 (0.93‐1.03)	.42	2813 (−7.8%)	0.92 (0.88‐0.97)	**.002**	2161 (−23.2%)	0.77 (0.73‐0.81)	**<.001**	2417 (+11.8%)	1.12 (1.06‐1.19)	**<.001**	2075 (−14.2%)	0.86 (0.81‐0.91)	**<.001**
*Facial soft tissue injuries*																
Northeast	26,798	34,256 (+27.8%)	1.28 (1.26‐1.30)	**<.001**	37,614 (+9.8%)	1.10 (1.08‐1.11)	**<.001**	30,140 (−19.9%)	0.80 (0.79‐0.81)	**<.001**	37,101 (+23.1%)	1.23 (1.21‐1.25)	**<.001**	39,802 (+7.3%)	1.07 (1.06‐1.09)	**<.001**
Midwest	22,787	22,661 (−0.6%)	0.99 (0.98‐1.01)	.55	24,317 (+7.3%)	1.07 (1.05‐1.09)	**<.001**	20,712 (−14.8%)	0.85 (0.84‐0.87)	**<.001**	23,145 (+11.7%)	1.12 (1.10‐1.14)	**<.001**	22,508 (−2.3%)	0.97 (0.95‐0.99)	**.003**
South	34,755	37,594 (+8.2%)	1.08 (1.07‐1.10)	**<.001**	39,682 (+5.6%)	1.06 (1.04‐1.07)	**<.001**	31,696 (−20.1%)	0.80 (0.79‐0.81)	**<.001**	36,771 (+16.0%)	1.16 (1.14‐1.18)	**<.001**	38,022 (+3.4%)	1.03 (1.02‐1.05)	**<.001**
West	18,280	21,240 (+16.2%)	1.16 (1.14‐1.19)	**<.001**	22,336 (+5.2%)	1.05 (1.03‐1.07)	**<.001**	19,444 (−13.0%)	0.87 (0.85‐0.89)	**<.001**	22,203 (+14.2%)	1.14 (1.12‐1.16)	**<.001**	22,431 (+1.0%)	1.01 (0.99‐1.03)	.28
Other/unknown	21,555	19,161 (−11.1%)	0.89 (0.87‐0.91)	**<.001**	18,108 (−5.5%)	0.95 (0.93‐0.96)	**<.001**	13,094 (−27.7%)	0.72 (0.71‐0.74)	**<.001**	14,631 (+11.7%)	1.12 (1.09‐1.14)	**<.001**	12,966 (−11.4%)	0.89 (0.87‐0.91)	**<.001**

Each RR (95% CI) and *P* value compares the given year to the year prior. Bold values are statistically significant.

Abbreviations: CI, confidence interval; RR, relative risk.

### Full Calendar Year Data

Supplemental Tables S2‐S5 and Figures S6‐S8, and S10, available online detail the incidence, mechanisms, and treatment of CMF fractures and soft tissue injuries for the full calendar years 2017 to 2022, reflecting similar trends to March to August.

## Discussion

This analysis of over 1,000,000 CMF fractures and soft tissue injuries across 83 HCOs highlights the COVID‐19 pandemic's significant impact. During March to August 2020, fractures decreased by −17.5% and soft tissue injuries by −19.0%. In 2021, there was a rebound with a +16.7% increase in fractures and +16.3% in soft tissue injuries compared to 2020. By 2022, fractures (−2.8%), soft tissue injuries (−4.5%), and operative repairs (−6.9% for fractures, −1.2% for soft tissue injuries) were slightly below prepandemic 2019 levels.

The observed national trends in CMF trauma are likely influenced by several factors. The pandemic's impact on behavior changes is evident in the significant reduction in facial trauma rates during the initial phase of the public health emergency, aligning with previous smaller‐scale findings.^7^, ^8^, ^9^, ^10^, ^11^, ^12^, ^13^, ^14^ Government‐imposed restrictions, such as shelter‐in‐place orders, travel advisories, and the closure of nonessential businesses likely played a crucial role in mitigating facial trauma incidents.^1^ The increased utilization of virtual platforms for work and school is also likely to have made a substantial contribution. Individuals may have also voluntarily refrained from leaving the safety of their homes to minimize the risk of contracting the virus.^21^ This was supported by a decline in the occurrence of falls, assaults, MVCs, pedestrian accidents, and athletic injuries at the height of the pandemic. However, not all injury mechanisms saw a decline during the pandemic. Injuries associated with bicycling, motorcycling, and off‐road vehicles actually increased in 2020, potentially due to an increased participation in these outdoor activities. Previous studies from various countries have presented varied results in trauma mechanism trends, though limited by small sample sizes.^7^, ^9^, ^10^, ^13^, ^22^, ^23^ Despite this, reports indicate increases in pedestrian versus automobile injuries,^7^ positive urine drug screens,^7^ falls,^9^ and domestic violence/assault/gun violence,^7^, ^9^, ^10^, ^13^, ^22^, ^23^ as well as decreased sporting injuries^9^ in 2020 compared to 2019. Our data aligns with some previous findings and contradicts others.

In this study, all regions of the United States saw significant reductions in CMF fractures and soft tissue injuries in 2020, though the Midwest and West experienced the smallest declines. The more rural areas in these regions,^24^ with less stringent lockdown measures and more opportunities for outdoor activities, may have contributed to these smaller reductions, compared to regions with stricter restrictions and higher population densities. Interestingly, despite notoriously relaxed lockdown measures in Southern states like Florida,^25^ this region, along with the Northeast, saw the most significant decreases in traumatic injuries in 2020.

A pronounced resurgence in the rate of facial trauma occurred in the postpandemic period in 2021. As society gradually returned to a semblance of normalcy, people may have resumed certain prepandemic behaviors and activities that carry a higher risk of injury. The relaxation of lockdowns and restrictions presumably led to increased travel, social interactions, and participation in events, contributing to a rise in traumatic incidents. These encompass common causes of facial trauma, including MVCs, violence, falls, sporting accidents, and work‐related injuries.^26^, ^27^

In 2022, the overall rates of CMF injuries were slightly lower compared to those observed in 2019 just before the onset of the pandemic. This may be due to continued adaptations in lifestyle, work, and educational practices that emerged during the pandemic, such as hybrid in‐office and remote employment.^28^ However, this trend was not universal across all age groups; there were declines in injuries among younger and middle‐aged individuals, while an increase in injuries was noted among the elderly in 2022 compared to prepandemic levels. One hypothesis for this discrepancy is that the reduced injury rates in the younger and middle‐aged groups might be linked to the increased implementation of the aforementioned lifestyle changes. It must also be noted that the prepandemic comparison group was deliberately limited to the year 2019 to capture the immediate prepandemic period. However, there was a consistent rise in trauma volumes from 2017 to 2019. Consequently, the overall rates of traumatic injuries in 2022, while lower than those in 2019, matched or exceeded the figures recorded in 2017 and 2018. The increase in trauma volumes during 2017 to 2019 was greater than that which could be attributed to population growth, as the US population grew by about 1% during that period.^29^ One likely contributing factor to this observed increase was the transition from the ICD‐9 to ICD‐10 diagnosis coding system in October 2015. The ICD‐10 system introduced new, specific codes for various facial trauma. Although TriNetX converts ICD‐9 to ICD‐10 codes when possible, it primarily relies on ICD‐10 coding. In the clinical setting, the transition to ICD‐10 likely required time for full implementation and accurate adoption across health care systems. Increasingly improved documentation and injury reporting practices in the following years may have contributed to the observed increase in trauma volumes in 2017‐2019, rather than reflecting a true increase in injury rates.

Demographic patterns revealed that young adult males aged 20 to 29 years were consistently most prone to CMF injuries, aligning with prior literature.^27^, ^30^ Notably, adolescents aged 10 to 14 saw the steepest declines in CMF fractures (−40%) and soft tissue injuries (−30%) during the pandemic, likely due to lifestyle changes from school closures and restricted extracurricular activities. A substantial body of research has indicated a rise in sedentary behavior and screen time, coupled with a decline in physical activity among adolescents during the pandemic.^31^, ^32^ Although this undoubtedly had negative impacts on overall health, the altered daily routines may have inadvertently created an environment with fewer opportunities for accidents or injuries. In contrast, the relatively modest decreases of −7% for fractures and −10% for soft tissue injuries observed among individuals in the 40 to 44 age group during the pandemic may be attributed to their potential engagement in essential activities that carried a persistent risk of injuries. It is conceivable that individuals in this age range, often managing work, family responsibilities, and potentially fulfilling essential roles, continued to navigate environments where injury risks were not as effectively mitigated by lockdown measures.^33^, ^34^ Additionally, elderly patients aged 75 to 79, 80 to 84, and 85+ experienced significant −20% reductions in both fractures and soft tissue injuries in 2020. This decline is particularly noteworthy given the increased mortality rates and higher levels of isolation faced by these age groups during the pandemic.

Concerning gender differences, despite more injuries among males overall, both genders saw similar declines in fractures and soft tissue injuries in 2020. This parallel reduction may stem from government restrictions, lifestyle changes, and altered daily activities affecting males and females equally.

Surgical trends closely followed injury patterns, with a decrease of over −20% for fractures and −12% for soft tissue injuries in 2020, followed by near‐return to prepandemic levels in 2021 and 2022. Nasal bone surgeries saw the most significant reduction in 2020, possibly due to fewer elective procedures during the peak of the pandemic.^35^ Among soft tissue injuries, adjacent tissue transfer showed the largest decrease in 2020 compared to less complex repairs, reflecting a possible shift towards conservative management when possible during the pandemic influenced by health care resource allocation and patient preferences.^36^

This study is the largest of its kind and the first to analyze postpandemic trends, but it has limitations. Data accuracy from TriNetX relies on the quality of electronic medical records, and the aforementioned transition from ICD‐9 to ICD‐10 may have affected the completeness of early data. Analyses are also limited by the specificity of existing diagnosis and procedure codes. Additionally, while data aggregation ensures patient confidentiality, the lack of detailed patient‐level data restricts thorough subgroup analyses. Although TriNetX provides regional information, it does not provide enough location detail to correlate HCOs with their local COVID‐19 restrictions. Therefore, as mentioned in the methods, the choice of the March to August timeframe as the “peak of the pandemic” was an adequate but imperfect representation.^16^, ^17^, ^18^ Another limitation of TriNetX is that its data is predominantly sourced from the Northeast and South, which limits broader geographic representation. Lastly, the study does not account for changes in health care‐seeking behavior, which may result in underestimating injury rates if some patients with minor injuries did not seek care during the pandemic.^37^

## Conclusion

CMF fractures and soft tissue injuries dropped significantly during the pandemic peak but rebounded toward prepandemic levels. Injury patterns varied over time, with young adult males being most affected and adolescents and the elderly showing the sharpest declines in 2020. Surgical trends mirrored these patterns, with the most significant reductions in nasal bone repairs and tissue transfers during the pandemic. This study furthers our understanding of COVID‐19's impact on CMF trauma.

## Author Contributions

**F. Jeffrey Lorenz**, concept design, data collection, reviewing data analysis, writing manuscript, presentation; **Andrew J. Rothka**, concept design, reviewing data analyses, critical editing of manuscript and final approval; **Heather K. Schopper**, concept design, reviewing data analyses, critical editing of manuscript and final approval; **Jessyka G. Lighthall**, concept design, reviewing data analyses, critical editing of manuscript and final approval.

## Disclosures

### Competing interests

The authors report no conflicts of interest.

### Funding source

The project was supported by the National Center for Advancing Translational Sciences, National Institutes of Health (NIH), through Grant UL1 TR002014.

## Supporting information

Supporting information.
