# Supplementary material for: Impact of the COVID‐19 Pandemic on the Incidence, Etiology, Demographics, and Treatment of Craniomaxillofacial Trauma
Source: Otolaryngol Head Neck Surg. 2024 Oct 1;172(2):444–56. doi: 10.1002/ohn.981 (PMC11773430; doi:10.1002/ohn.981)
Supplement: Supplementary file 1 — Supporting information. [file OHN-172-444-s001.docx]

**Supplement 1: Diagnosis (ICD-10) and Procedure (CPT) Codes Utilized to Conduct Analysis**

Facial Fractures:

S02.0 Fracture of vault of skull

S02.1 Fracture of base of skull

S02.2 Fracture of nasal bones

S02.3 Fracture of orbital floor

S02.4 Fracture of malar, maxillary, and zygoma bones

S02.6 Fracture of mandible

S02.8 Fractures of other specified skull and facial bones

S02.9 Fracture of unspecified skull and facial bones

Facial Fracture Operative Repair:

1003921 Closed treatment of nasal bone fracture with manipulation

1003924 Open treatment of nasal fracture

1003930 Open treatment of nasoethmoid fracture

1003938 Open treatment of nasomaxillary complex fracture (LeFort II type)

1003945 Open treatment of complicated (e.g., comminuted or involving cranial nerve foramina) fractures of malar area, including zygomatic arch and malar tripod

1003948 Open treatment of orbital floor blowout fracture

1003954 Closed treatment of fracture of orbit, except blowout

1003957 Open treatment of fracture of orbit, except blowout

1003965 Open treatment of craniofacial separation (LeFort III type)

1003972 Closed treatment of mandibular fracture

1003978 Open treatment of mandibular fracture

1003983 Closed treatment of temporomandibular dislocation

1014057 Open treatment of palatal or maxillary fracture (LeFort I type)

21336 Open treatment of nasal septal fracture, with or without stabilization

21337 Closed treatment of nasal septal fracture, with or without stabilization

21340 Percutaneous treatment of nasoethmoid complex fracture, with splint, wire or head Fixation, including repair of canthal ligaments and/or the nasolacrimal apparatus

21343 Open treatment of depressed frontal sinus fracture

21344 Open treatment of complicated (e.g., comminuted or involving posterior wall) frontal sinus fracture, via coronal or multiple approaches

21344 Open treatment of complicated (e.g., comminuted or involving posterior wall) frontal sinus fracture, via coronal or multiple approaches

21345 Closed treatment of nasomaxillary complex fracture (LeFort II type), with interdental wire fixation or fixation of denture or splint

21355 Percutaneous treatment of fracture of malar area, including zygomatic arch and malar tripod, with manipulation

21356 Open treatment of depressed zygomatic arch fracture (e.g., Gillies approach)

21360 Open treatment of depressed malar fracture, including zygomatic arch and malar tripod

21421 Closed treatment of palatal or maxillary fracture (LeFort I type), with interdental wire fixation or fixation of denture or splint

21431 Closed treatment of craniofacial separation (LeFort III type) using interdental wire fixation of denture or splint

21440 Closed treatment of mandibular or maxillary alveolar ridge fracture (separate procedure)

21445 Open treatment of mandibular or maxillary alveolar ridge procedure (separate procedure)

21452 Percutaneous treatment of mandibular fracture, with external fixation

21453 Closed treatment of mandibular fracture with interdental fixation

21454 Open treatment of mandibular fracture with external fixation

21465 Open treatment of mandibular condylar fracture

21470 Open treatment of complicated mandibular fracture by multiple surgical approaches including internal fixation, interdental fixation, and/or wiring of dentures or splints

21490 Open treatment of temporomandibular dislocation

Soft Tissue Injuries:

S00.01 Abrasion of scalp

S00.21 Abrasion of eyelid and periocular area

S00.31 Abrasion of nose

S00.41 Abrasion of ear

S00.51 Abrasion of lip and oral cavity

S00.81 Abrasion of other part of head

S00.91 Abrasion of unspecified part of head

S10.91 Abrasion of unspecified part of neck

S01.0 Open wound of scalp

S01.1 Open wound of eyelid and periocular area

S01.2 Open wound of nose

S01.3 Open wound of ear

S01.4 Open wound of cheek and temporomandibular area

S01.5 Open wound of lip and oral cavity

S01.8 Open wound of other parts of head

S01.9 Open wound of unspecified part of head

S11.8 Open wound of other specified parts of neck

S11.9 Open wound of unspecified part of neck

Soft Tissue Repair:

1003310 Simple repair of superficial wounds of scalp, neck, axillae, external genitalia, trunk, and/or extremities (including hands and feet)

1003317 Simple repair of superficial wounds of face, ears, eyelids, nose, lips and/or mucous membranes

1003329 Repair, intermediate, wounds of scalp, axillae, trunk, and/or extremities (excluding hands and feet

1003336 Repair, intermediate, wounds of neck, hands, feet and/or external genitalia

1003343 Repair, intermediate, wounds of face, ears, eyelids, nose, lips and/or mucous membranes

1003356 Repair, complex, scalp, arms, and/or legs

1003360 Repair, complex, forehead, cheeks, chin, mouth, neck, axillae, genitalia, hands and/or feet

1003364 Repair, complex, eyelids, nose, ears and/or lips

1003374 Adjacent tissue transfer or rearrangement, scalp, arms, and/or legs

1003377 Adjacent tissue transfer or rearrangement, forehead, cheeks, chin, mouth, neck, axillae, genitalia, hands, and/or feet

1003380 Adjacent tissue transfer or rearrangement, eyelids, nose, ears, and/or lips

Injury Mechanisms

V86 Occupant of special all-terrain or other offroad motor vehicle, injured and transport accident

V40–V49 Car occupant injured and transport accident

V50–V59 Occupant of pickup truck or van injured and transport accident

V60–V69 Occupant of heavy transport vehicle injured and transport accident

V70–V79 Bus occupant injured and transport accident

V82 Occupant of powered street car injured and transport accident

V87 Traffic accident unspecified type but victims mode of transport unknown

V89 Motor or nonmotor vehicle accident, type of vehicle unspecified

V00–V09 Pedestrian injured in transport accident

V10–V19 Pedal cycle rider injured and transport accident

V20–V29 Motorcycle rider injured in transport accident

W21 Striking against or struck by sports equipment

Y92.3 Sports and athletics area as the place of occurrence of the external cause

X71–X83 Intentional self-harm

W00–W19 Slipping, tripping, stumbling and falls

W54.0 Bitten by dog

X92–Y09 Assault

Supplement 2: Craniomaxillofacial Fracture and Soft Tissue Injury Volumes During Entire Calendar Years Before, During, and After the COVID-19 Pandemic (January-December 2017-2022)

| Fracture Site | 2017 | 2018 (% Change) | RR (95% CI) | P-Value | 2019 (% Change) | RR (95% CI) | P-Value | 2020 (% Change) | RR (95% CI) | P-Value | 2021 (% Change) | RR (95% CI) | P-Value | 2022 (% Change) | RR (95% CI) | P-Value |
| --- | --- | --- | --- | --- | --- | --- | --- | --- | --- | --- | --- | --- | --- | --- | --- | --- |
| **Craniomaxillofacial Fractures** | | | | | | | | | | | | | | | | |
| Total Patients with Fractures | 69,871 | 74,769 (+7.0%) | 1.07 (1.06-1.08) | **<0.001** | 76,991 (+3.0%) | 1.03 (1.02-1.04) | **<0.001** | 68,159 (-11.5%) | 0.89 (0.88-0.89) | **<0.001** | 73,492 +7.8%) | 1.08 (1.07-1.09) | **<0.001** | 74,391 (+1.2%) | 1.01 (1.00-1.02) | **0.02** |
| Vault of Skull | 16,543 | 17,654 (+6.7%) | 1.07 (1.04-1.09) | **<0.001** | 17,078 (-3.3%) | 0.97 (0.95-0.99) | **0.002** | 14,832 (-13.2%) | 0.87 (0.85-0.89) | **<0.001** | 14,741 (-0.6%) | 0.99 (0.97-1.02) | 0.60 | 13,494 (-8.5%) | 0.92 (0.89-0.94) | **<0.001** |
| Base of Skull | 21,580 | 22,468 (+4.1%) | 1.04 (1.02-1.06) | **<0.001** | 22,206 (-1.2%) | 0.99 (0.97-1.01) | 0.22 | 19,863 (-10.6%) | 0.89 (0.88-0.91) | **<0.001** | 19,913 (+0.3%) | 1.00 (0.98-1.02) | 0.80 | 18,413 (-7.5%) | 0.92 (0.91-0.94) | **<0.001** |
| Nasal Bones | 36,843 | 40,296 (+9.4%) | 1.09 (1.08-1.11) | **<0.001** | 41,809 (+3.8%) | 1.04 (1.02-1.05) | **<0.001** | 35,861 (-14.2%) | 0.86 (0.85-0.87) | **<0.001** | 39,283 (+9.5%) | 1.10 (1.08-1.11) | **<0.001** | 39,325 (+0.1%) | 1.00 (0.99-1.02) | 0.88 |
| Orbital Floor | 20,408 | 21,787 (+6.8%) | 1.07 (1.05-1.09) | **<0.001** | 21,620 (-0.8%) | 0.99 (0.97-1.01) | 0.42 | 18,605 (-13.9%) | 0.86 (0.84-0.88) | **<0.001** | 18,708 (+0.6%) | 1.01 (0.99-1.03) | 0.59 | 17,528 (-6.3%) | 0.94 (0.92-0.96) | **<0.001** |
| Malar, Maxillary, and Zygoma | 23,542 | 24,924 (+5.9%) | 1.06 (1.04-1.08) | **<0.001** | 25,051 (+0.5%) | 1.01 (0.99-1.02) | 0.57 | 22,045 (-12.0%) | 0.88 (0.86-0.90) | **<0.001** | 22,333 (+1.3%) | 1.01 (0.99-1.03) | 0.17 | 21,307 (-4.6%) | 0.95 (0.94-0.97) | **<0.001** |
| Mandible | 18,961 | 19,979 (+5.4%) | 1.05 (1.03-1.07) | **<0.001** | 19,425 (-2.8%) | 0.97 (0.95-0.99) | **0.01** | 16,501 (-15.1%) | 0.85 (0.83-0.87) | **<0.001** | 16,550 (+0.3%) | 1.00 (0.98-1.02) | 0.79 | 15,248 (-7.9%) | 0.92 (0.90-0.94) | **<0.001** |
| Other Skull and Facial Bones | 29,438 | 30,906 (+5.0%) | 1.05 (1.03-1.07) | **<0.001** | 30,908 (+0.0%) | 1.00 (0.98-1.02) | 0.99 | 27,749 (-10.2%) | 0.9 (0.88-0.91) | **<0.001** | 28,872 (+4.0%) | 1.04 (1.02-1.06) | **<0.001** | 27,804 (-3.7%) | 0.96 (0.95-0.98) | **<0.001** |
| Mean Fractures per Patient | 2.39 | 2.38 (-0.4%) | - | **-** | 2.31 (-3.0%) | - | - | 2.28 (-1.3%) | - | **-** | 2.18 (-4.4%) | - | **-** | 2.06 (-5.5%) | - | **-** |
| **Facial Soft Tissue Injuries** | | | | | | | | | | | | | | | | |
| Total Patients with Soft Tissue Injuries | 243,170 | 265,394 (+9.1%) | 1.09 (1.09-1.10) | **<0.001** | 279,361 (+5.3%) | 1.05 (1.05-1.06) | **<0.001** | 243,869 (-12.7%) | 0.87 (0.87-0.88) | **<0.001** | 264,940 (+8.6%) | 1.09 (1.08-1.09) | **<0.001** | 265,801 (+0.3%) | 1.00 (1.00-1.01) | 0.24 |
| Scalp | 61,522 | 69,655 (+13.2%) | 1.13 (1.12-1.14) | **<0.001** | 74,406 (+6.8%) | 1.07 (1.06-1.08) | **<0.001** | 66,734 (-10.3%) | 0.9 (0.89-0.91) | **<0.001** | 72,009 (+7.9%) | 1.08 (1.07-1.09) | **<0.001** | 73,236 (+1.7%) | 1.02 (1.01-1.03) | **0.001** |
| Eyelid and Periocular Area | 33,839 | 37,253 (+10.1%) | 1.10 (1.08-1.12) | **<0.001** | 38,907 (+4.4%) | 1.04 (1.03-1.06) | **<0.001** | 34,343 (-11.7%) | 0.88 (0.87-0.90) | **<0.001** | 37,634 (+9.6%) | 1.10 (1.08-1.11) | **<0.001** | 38,267 (+1.7%) | 1.02 (1.00-1.03) | **0.02** |
| Nose | 15,495 | 17,607 (+13.6%) | 1.14 (1.11-1.16) | **<0.001** | 19,048 (+8.2%) | 1.08 (1.06-1.10) | **<0.001** | 17,000 (-10.8%) | 0.89 (0.87-0.91) | **<0.001** | 18,366 (+8.0%) | 1.08 (1.06-1.10) | **<0.001** | 18,736 (+2.0%) | 1.02 (1.00-1.04) | **0.05** |
| Ear | 14,140 | 16,354 (+15.7%) | 1.16 (1.13-1.18) | **<0.001** | 17,978 (+9.9%) | 1.10 (1.08-1.12) | **<0.001** | 15,602 (-13.2%) | 0.87 (0.85-0.89) | **<0.001** | 18,180 (+16.5%) | 1.17 (1.14-1.19) | **<0.001** | 18,198 (+0.1%) | 1.00 (0.98-1.02) | 0.92 |
| Cheek and Temporomandibular Area | 7,939 | 8,338 (+5.0%) | 1.05 (1.02-1.08) | **0.002** | 9,087 (+9.0%) | 1.09 (1.06-1.12) | **<0.001** | 8,586 (-5.5%) | 0.94 (0.92-0.97) | **<0.001** | 8,823 (+2.8%) | 1.03 (1.00-1.06) | **0.07** | 9,092 (+3.0%) | 1.03 (1.00-1.06) | **0.04** |
| Lip and Oral Cavity | 40,915 | 45,582 (+11.4%) | 1.11 (1.10-1.13) | **<0.001** | 48,162 (+5.7%) | 1.06 (1.04-1.07) | **<0.001** | 42,115 (-12.6%) | 0.87 (0.86-0.89) | **<0.001** | 44,922 (+6.7%) | 1.07 (1.05-1.08) | **<0.001** | 44,279 (-1.4%) | 0.99 (0.97-1.00) | **0.03** |
| Other Part of Head | 120,169 | 131,274 (+9.2%) | 1.09 (1.08-1.10) | **<0.001** | 139,543 (+6.3%) | 1.06 (1.06-1.07) | **<0.001** | 122,672 (-12.1%) | 0.88 (0.87-0.89) | **<0.001** | 132,496 (+8.0%) | 1.08 (1.07-1.09) | **<0.001** | 132,811 (+0.2%) | 1.00 (0.99-1.01) | 0.54 |
| Neck | 8,768 | 9,945 (+13.4%) | 1.13 (1.10-1.17) | **<0.001** | 10,628 (+6.9%) | 1.07 (1.04-1.10) | **<0.001** | 9,525 (-10.4%) | 0.9 (0.87-0.92) | **<0.001** | 10,337 (+8.5%) | 1.09 (1.06-1.12) | **<0.001** | 10,120 (-2.1%) | 0.98 (0.95-1.01) | 0.13 |
| Mean Soft Tissue Injuries per Patient | 1.25 | 1.27 (+1.6%) | - | **-** | 1.28 (+0.8%) | - | **-** | 1.30 (+1.6%) | - | **-** | 1.29 (-0.8%) | - | **-** | 1.30 (+0.8%) | - | - |

Abbreviations: RR, relative risk; CI, confidence interval.

Each RR (95% CI) and P value compares the given year to the year prior.

Please note individual CMF fracture and soft tissue injury counts exceed total patients with fractures and soft tissue injuries given the fact that some patients had multiple injuries.

Supplement 3: Craniomaxillofacial Fracture and Soft Tissue Injury Mechanisms During Entire Calendar Years Before, During, and After the COVID-19 Pandemic (January-December 2017-2022)

| Injury Mechanism | 2017 | 2018 (% Change) | RR (95% CI) | P-Value | 2019 (% Change) | RR (95% CI) | P-Value | 2020 (% Change) | RR (95% CI) | P-Value | 2021 (% Change) | RR (95% CI) | P-Value | 2022 (% Change) | RR (95% CI) | P-Value |
| --- | --- | --- | --- | --- | --- | --- | --- | --- | --- | --- | --- | --- | --- | --- | --- | --- |
| Falls | 83,147 | 96,415 (+16.0%) | 1.16 (1.15-1.17) | **<0.001** | 106,393 (+10.3%) | 1.10 (1.09-1.11) | **<0.001** | 94,070 (-11.6%) | 0.88 (0.88-0.89) | **<0.001** | 100,376 (+6.7%) | 1.07 (1.06-1.08) | **<0.001** | 105,063 (+4.7%) | 1.05 (1.04-1.06) | **<0.001** |
| Assault | 24,040 | 25,988 (+8.1%) | 1.08 (1.06-1.10) | **<0.001** | 26,772 (+3.0%) | 1.03 (1.01-1.05) | **<0.001** | 23,808 (-11.1%) | 0.89 (0.87-0.90) | **<0.001** | 23,292 (-2.2%) | 0.98 (0.96-1.00) | **0.02** | 22,698 (-2.6%) | 0.97 (0.96-0.99) | **0.01** |
| MVCs | 25,248 | 27,165 (+7.6%) | 1.08 (1.06-1.09) | **<0.001** | 28,639 (+5.4%) | 1.05 (1.04-1.07) | **<0.001** | 27,326 (-4.6%) | 0.95 (0.94-0.97) | **<0.001** | 30,564 (+11.8%) | 1.12 (1.10-1.14) | **<0.001** | 29,213 (-4.4%) | 0.96 (0.94-0.97) | **<0.001** |
| Dog Bite | 6,135 | 7,097 (+15.7%) | 1.16 (1.12-1.20) | **<0.001** | 7,738 (+9.0%) | 1.09 (1.06-1.13) | **<0.001** | 8,246 (+6.6%) | 1.07 (1.03-1.10) | **<0.001** | 8,555 (+3.7%) | 1.04 (1.01-1.07) | **0.02** | 8,542 (-0.2%) | 1.00 (0.97-1.03) | 0.92 |
| Pedestrian | 5,119 | 5,564 (+8.7%) | 1.09 (1.05-1.13) | **<0.001** | 5,976 (+7.4%) | 1.07 (1.04-1.11) | **<0.001** | 5,669 (-5.1%) | 0.95 (0.91-0.98) | **0.004** | 6,607 (+16.5%) | 1.17 (1.12-1.21) | **<0.001** | 6,550 (-0.9%) | 0.99 (0.96-1.03) | 0.62 |
| Bike Accident | 5,444 | 5,531 (+1.6%) | 1.02 (0.98-1.05) | 0.41 | 5,710 (+3.2%) | 1.03 (0.99-1.07) | 0.09 | 6,608 (+15.7%) | 1.16 (1.12-1.20) | **<0.001** | 5,382 (-18.6%) | 0.81 (0.79-0.84) | **<0.001** | 5,085 (-5.5%) | 0.94 (0.91-0.98) | **0.004** |
| Motorcycle | 3,052 | 3,292 (+7.9%) | 1.08 (1.03-1.13) | **0.003** | 3,208 (-2.6%) | 0.97 (0.93-1.02) | 0.30 | 3,586 (+11.8%) | 1.12 (1.07-1.17) | **<0.001** | 3,975 (+10.8%) | 1.11 (1.06-1.16) | **<0.001** | 3,986 (+0.3%) | 1.00 (0.96-1.05) | 0.90 |
| Self-Harm | 1,771 | 1,987 (+12.2%) | 1.12 (1.05-1.20) | **<0.001** | 1,923 (-3.2%) | 0.97 (0.91-1.03) | 0.31 | 1,951 (+1.5%) | 1.01 (0.95-1.08) | 0.65 | 1,915 (-1.8%) | 0.98 (0.92-1.05) | 0.56 | 1,909 (-0.3%) | 1.00 (0.94-1.06) | 0.92 |
| Athletic Injury | 3,728 | 3,964 (+6.3%) | 1.06 (1.02-1.11) | **0.01** | 3,785 (-4.5%) | 0.95 (0.91-1.00) | **0.04** | 2,135 (-43.6%) | 0.56 (0.53-0.59) | **<0.001** | 3,038 (+42.3%) | 1.42 (1.35-1.50) | **<0.001** | 3,339 (+9.9%) | 1.10 (1.05-1.15) | **<0.001** |
| Off-Road Vehicles | 2,016 | 2,088 (+3.6%) | 1.04 (0.97-1.10) | 0.26 | 2,325 (+11.4%) | 1.11 (1.05-1.18) | **<0.001** | 3,375 (+45.2%) | 1.45 (1.38-1.53) | **<0.001** | 3,071 (-9.0%) | 0.91 (0.87-0.96) | **<0.001** | 2,886 (-6.0%) | 0.94 (0.89-0.99) | **0.02** |

Abbreviations: RR, relative risk; CI, confidence interval.

Each RR (95% CI) and P value compares the given year to the year prior.

Please note mechanism data does not add up to total injuries given the fact that some patients did not have their mechanism coded or had uncommon causes which were not included in the analysis.

Supplement 4: Craniomaxillofacial Fracture Operative Repair Volumes During Entire Calendar Years Before, During, and After the COVID-19 Pandemic (January-December 2017-2022)

| Surgery Type | 2017 | 2018 (% Change) | RR (95% CI) | P-Value | 2019 (% Change) | RR (95% CI) | P-Value | 2020 (% Change) | RR (95% CI) | P-Value | 2021 (% Change) | RR (95% CI) | P-Value | 2022 (% Change) | RR (95% CI) | P-Value |
| --- | --- | --- | --- | --- | --- | --- | --- | --- | --- | --- | --- | --- | --- | --- | --- | --- |
| **Craniomaxillofacial Fracture Operative Repairs** | | | | | | | | | | | | | | | | |
| Total Patients with Surgery | 7,538 | 8,064 (+7.0%) | 1.07 (1.04-1.10) | **<0.001** | 8,064 (+0.0%) | 1.00 (0.97-1.03) | 1.0 | 6,898 (-14.5%) | 0.86 (0.83-0.88) | **<0.001** | 7,232 (4.8%) | 1.05 (1.01-1.08) | **0.004** | 7,396 (+2.3%) | 1.02 (0.99-1.06) | 0.18 |
| Nasal Bone Surgery | 2,936 | 3,086 (+5.1%) | 1.05 (1.00-1.11) | **0.05** | 3,053 (-1.1%) | 0.99 (0.94-1.04) | 0.67 | 2,511 (-17.8%) | 0.82 (0.78-0.87) | **<0.001** | 2,825 (+12.5%) | 1.13 (1.07-1.19) | **<0.001** | 3,100 (+9.7%) | 1.10 (1.04-1.15) | **<0.001** |
| Mandible Surgery | 3,160 | 3,384 (+7.1%) | 1.07 (1.02-1.12) | **0.01** | 3,324 (-1.8%) | 0.98 (0.94-1.03) | 0.46 | 2,910 (-12.5%) | 0.88 (0.83-0.92) | **<0.001** | 2,983 (+2.5%) | 1.03 (0.97-1.08) | 0.34 | 2,963 (-0.7%) | 0.99 (0.94-1.05) | 0.80 |
| Midface Surgery | 1,651 | 1,573 (-4.7%) | 0.95 (0.89-1.02) | 0.17 | 1,456 (-7.4%) | 0.93 (0.86-0.99) | **0.03** | 1,327 (-8.9%) | 0.91 (0.85-0.98) | **0.01** | 1,283 (-3.3%) | 0.97 (0.90-1.04) | 0.39 | 1,290 (+0.5%) | 1.01 (0.93-1.09) | 0.89 |
| Frontal Bone Surgery | 125 | 139 (+11.2%) | 1.11 (0.87-1.42) | 0.39 | 136 (-2.2%) | 0.98 (0.77-1.24) | 0.86 | 146 (+7.4%) | 1.07 (0.85-1.36) | 0.55 | 129 (-11.6%) | 0.88 (0.70-1.12) | 0.31 | 119 (-7.8%) | 0.92 (0.72-1.18) | 0.53 |
| Orbital Surgery | 1,621 | 1,741 (+7.4%) | 1.07 (1.00-1.15) | **0.04** | 1,679 (-3.6%) | 0.96 (0.90-1.03) | 0.29 | 1,347 (-19.8%) | 0.8 (0.75-0.86) | **<0.001** | 1,327 (-1.5%) | 0.99 (0.91-1.06) | 0.70 | 1,231 (-7.2%) | 0.93 (0.86-1.00) | 0.06 |
| Mean Procedures per Patient | 1.26 | 1.23 (-2.4%) | - | **-** | 1.20 (-2.4%) | - | - | 1.19 (-0.8%) | - | **-** | 1.18 (-0.9%) | - | - | 1.18 (0%) | - | **-** |
| **Facial Soft Tissue Repairs** | | | | | | | | | | | | | | | | |
| Total Patients with Soft Tissue Repair | 103,226 | 113,486 (+9.9%) | 1.10 (1.09-1.11) | **<0.001** | 121,367 (+6.9%) | 1.07 (1.06-1.08) | **<0.001** | 113,209 (-6.7%) | 0.93 (0.93-0.94) | **<0.001** | 121,877 (+7.7%) | 1.08 (1.07-1.09) | **<0.001** | 119,319 (-2.1%) | 0.98 (0.97-0.99) | **<0.001** |
| Simple Repair | 88,431 | 96,305 (+8.9%) | 1.09 (1.08-1.10) | **<0.001** | 103,449 (+7.4%) | 1.07 (1.06-1.08) | **<0.001** | 96,106 (-7.1%) | 0.93 (0.92-0.94) | **<0.001** | 104,120 (+8.3%) | 1.08 (1.07-1.09) | **<0.001** | 103,097 (-1.0%) | 0.99 (0.98-1.00) | **0.02** |
| Intermediate Repair | 13,205 | 15,382 (+16.5%) | 1.16 (1.14-1.19) | **<0.001** | 16,143 (+4.9%) | 1.05 (1.03-1.07) | **<0.001** | 15,568 (-3.6%) | 0.96 (0.94-0.99) | **0.001** | 16,113 (+3.5%) | 1.04 (1.01-1.06) | **0.002** | 15,144 (-6.0%) | 0.94 (0.92-0.96) | **<0.001** |
| Complex Repair | 6,545 | 6,712 (+2.6%) | 1.03 (0.99-1.06) | 0.15 | 6,873 (+2.4%) | 1.02 (0.99-1.06) | 0.17 | 6,422 (-6.6%) | 0.93 (0.90-0.97) | **<0.001** | 6,145 (-4.3%) | 0.96 (0.92-0.99) | **0.01** | 5,785 (-5.9%) | 0.94 (0.91-0.98) | **<0.001** |
| Adjacent Tissue Transfer or Rearrangement | 2,092 | 2,470 (+18.1%) | 1.18 (1.11-1.25) | **<0.001** | 2,498 (+1.1%) | 1.01 (0.96-1.07) | 0.69 | 2,233 (-10.6%) | 0.89 (0.84-0.95) | **<0.001** | 2,390 (+7.0) | 1.07 (1.01-1.13) | **0.02** | 2,191 (-8.3%) | 0.92 (0.87-0.97) | **0.003** |
| Mean Procedures per Patient | 1.07 | 1.07 (0%) | - | **-** | 1.06 (-0.9%) | - | - | 1.06 (0%) | - | **-** | 1.06 (0%) | - | - | 1.06 (0%) | - | **-** |

Abbreviations: RR, relative risk; CI, confidence interval.

Each RR (95% CI) and P value compares the given year to the year prior.

Please note individual surgeries and repairs exceed total patients with surgery or soft tissue repairs given the fact that some patients had multiple injuries repaired.

Supplement 5: Craniomaxillofacial Fracture and Soft Tissue Injury Volumes During Entire Calendar Years Before, During, and After the COVID-19 Pandemic (January-December 2017-2022), Stratified by Region

| Region | 2017 | 2018 (% Change) | RR (95% CI) | P-Value | 2019 (% Change) | RR (95% CI) | P-Value | 2020 (% Change) | RR (95% CI) | P-Value | 2021 (% Change) | RR (95% CI) | P-Value | 2022 (% Change) | RR (95% CI) | P-Value |  |
| --- | --- | --- | --- | --- | --- | --- | --- | --- | --- | --- | --- | --- | --- | --- | --- | --- | --- |
| **Craniomaxillofacial Fractures** | | | | | | | | | | | | | | | | | |
| Northeast | 21,811 | 24,943 (+14.4%) | 1.14 (1.12-1.16) | **<0.001** | 26,210 (+5.1%) | 1.05 (1.03-1.07) | **<0.001** | 22,613 (-13.7%) | 0.86 (0.85-0.88) | **<0.001** | 24,880 (+10.0%) | 1.10 (1.08-1.12) | **<0.001** | 25,797 (+3.7%) | 1.04 (1.02-1.06) | **<0.001** |  |
| Midwest | 11,523 | 11,501 (-0.2%) | 1.0 (0.97-1.02) | 0.88 | 11,764 (+2.3%) | 1.02 (1.0-1.05) | 0.08 | 11,298 (-4.0%) | 0.96 (0.94-0.99) | **0.002** | 11,827 (+4.7%) | 1.05 (1.02-1.07) | **<0.001** | 11,235 (-5.0%) | 0.95 (0.93-0.97) | **<0.001** |  |
| South | 21,686 | 22,482 (+3.7%) | 1.04 (1.02-1.06) | **<0.001** | 23,236 (+3.4%) | 1.03 (1.01-1.05) | **<0.001** | 20,436 (-12.1%) | 0.88 (0.86-0.90) | **<0.001** | 21,662 (+6.0%) | 1.06 (1.04-1.08) | **<0.001** | 22,830 (+5.4%) | 1.05 (1.03-1.07) | **<0.001** |  |
| West | 8,840 | 9,975 (+12.8%) | 1.13 (1.10-1.16) | **<0.001** | 10,214 (+2.4%) | 1.02 (1.0-1.05) | 0.09 | 9,222 (9.7%) | 0.90 (0.88-0.93) | **<0.001** | 10,311 (+11.8%) | 1.12 (1.09-1.15) | **<0.001** | 10,365 (+0.5%) | 1.01 (0.98-1.03) | 0.71 |  |
| Other/Unknown | 6,011 | 5,868 (-2.4%) | 0.98 (0.94-1.01) | 0.19 | 5,567 (-5.1%) | 0.95 (0.91-0.98) | **0.005** | 4,590 (-17.6%) | 0.82 (0.79-0.86) | **<0.001** | 4,812 (+4.8%) | 1.05 (1.01-1.09) | **0.02** | 4,164 (-13.5%) | 0.87 (0.83-0.90) | **<0.001** |  |
| **Facial Soft Tissue Injuries** | | | | | | | | | | | | | | | | | |
| Northeast | 53,205 | 67,669 (+27.2%) | 1.27 (1.26-1.29) | **<0.001** | 73,801 (+9.1%) | 1.09 (1.08-1.10) | **<0.001** | 65,414 (-11.4%) | 0.89 (0.88-0.90) | **<0.001** | 74,120 (+13.3%) | 1.13 (1.12-1.15) | **<0.001** | 77,527 (+4.6%) | 1.05 (1.04-1.06) | **<0.001** |  |
| Midwest | 43,960 | 44,248 (+0.7%) | 1.01 (0.99-1.02) | 0.33 | 47,345 (+7.0%) | 1.07 (1.06-1.08) | **<0.001** | 43,511 (-8.1%) | 0.92 (0.91-0.93) | **<0.001** | 45,683 (+5.0%) | 1.05 (1.04-1.06) | **<0.001** | 44,456 (-2.7%) | 0.97 (0.96-0.99) | **<0.001** |  |
| South | 69,794 | 74,463 (+6.7%) | 1.07 (1.06-1.08) | **<0.001** | 78,642 (+5.6%) | 1.06 (1.05-1.07) | **<0.007** | 65,554 (-16.7%) | 0.83 (0.83-0.84) | **<0.001** | 70,208 (+7.1%) | 1.07 (1.06-1.08) | **<0.001** | 73,061 (+4.1%) | 1.04 (1.03-1.05) | **<0.001** |  |
| West | 36,239 | 42,019 (+15.9%) | 1.16 (1.14-1.18) | **<0.001** | 43,811 (+4.3%) | 1.04 (1.03-1.06) | **<0.001** | 40,827 (-6.8%) | 0.93 (0.92-0.94) | **<0.001** | 44,674 (+9.4%) | 1.09 (1.08-1.11) | **<0.001** | 44,911 (+0.5%) | 1.01 (0.99-1.02) | 0.43 |  |
| Other/Unknown | 39,972 | 36,995 (-7.5%) | 0.93 (0.91-0.94) | **<0.001** | 35,762 (-3.3%) | 0.97 (0.95-0.98) | **<0.001** | 28,563 (-20.1%) | 0.80 (0.79-0.81) | **<0.001** | 30,255 (+5.9%) | 1.06 (1.04-1.08) | **<0.001** | 25,846 (-14.6%) | 0.85 (0.84-0.87) | **<0.001** |  |

Abbreviations: RR, relative risk; CI, confidence interval.

Each RR (95% CI) and P value compares the given year to the year prior.

Supplement 6: Graphical Representation of Craniomaxillofacial A) Fracture and B) Soft Tissue Injury Volumes During Entire Calendar Years Before, During, and After the COVID-19 Pandemic (January-December 2017-2022)

1. B)

Supplement 7: Graphical Representation of Craniomaxillofacial Fracture and Soft Tissue Injury Mechanisms During Entire Calendar Years Before, During, and After the COVID-19 Pandemic (January-December 2017-2022)

Supplement 8: Graphical Representation of Craniomaxillofacial A) Fracture and B) Soft Tissue Injury Operative Repair Volumes During Entire Calendar Years Before, During, and After the COVID-19 (January-December 2017-2022)

1. B)

Supplement 9: Graphical Representation of Craniomaxillofacial A) Fracture and B) Soft Tissue Injury Volumes During March-August of Years Before, During, and After the COVID-19 Pandemic (2017-2022), Stratified by Region

A) B) Supplement 10: Graphical Representation of Craniomaxillofacial A) Fracture and B) Soft Tissue Injury Volumes During Entire Calendar Years Before, During, and After the COVID-19 Pandemic (January-December 2017-2022), Stratified by Region

A) B)
